# Allergic inflammation is initiated by IL-33–dependent crosstalk between mast cells and basophils

**DOI:** 10.1371/journal.pone.0226701

**Published:** 2020-01-15

**Authors:** Chia-Lin Hsu, Krishan D. Chhiba, Rebecca Krier-Burris, Shweta Hosakoppal, Sergejs Berdnikovs, Mendy L. Miller, Paul J. Bryce

**Affiliations:** Division of Allergy-Immunology, Department of Medicine, Feinberg School of Medicine, Northwestern University, Chicago, IL, United States of America; Toho University Graduate School of Medicine, JAPAN

## Abstract

IgE-primed mast cells in peripheral tissues, including the skin, lung, and intestine, are key initiators of allergen-triggered edema and inflammation. Particularly in severe forms of allergy, this inflammation becomes strongly neutrophil dominated, and yet how mast cells coordinate this type of response is unknown. We and others have reported that activated mast cells––a hematopoietic cell type––can produce IL-33, a cytokine known to participate in allergic responses but generally considered as being of epithelial origin and driving Type 2 immune responses (e.g., ILC2 and eosinophil activation). Using models of skin anaphylaxis, our data reveal that mast cell-derived IL-33 also initiates neutrophilic inflammation. We demonstrate a cellular crosstalk mechanism whereby activated mast cells crosstalk to IL-33 receptor–bearing basophils, driving these basophils to adopt a unique response signature rich in neutrophil-associated molecules. We further establish that basophil expression of CXCL1 is necessary for IgE-driven neutrophilic inflammation. Our findings thus unearth a new mechanism by which mast cells initiate local inflammation after antigen triggering and might explain the complex inflammatory phenotypes observed in severe allergic diseases. Moreover, our findings (i) establish a functional link from IL-33 to neutrophilic inflammation that extends IL-33–mediated biology well beyond that of Type 2 immunity, and (ii) demonstrate the functional importance of hematopoietic cell–derived IL-33 in allergic pathogenesis.

## Introduction

IgE-associated responses to allergens is a central initiating process in atopic diseases, including asthma, food allergy and urticarial reactions. While initial edematous responses are typically controlled through antihistamines, local inflammatory late-phase reactions occur in some cases, resulting in painful skin responses and impaired breathing when it occurs in the lung, although clinical heterogeneity in the magnitude of these responses is seen amongst patients [1]. Neutrophil infiltration is a hallmark of these late-phase reactions and is responsible for much of this inflammation. Previous studies show that tissue-resident mast cells are required for this neutrophilic infiltration to occur [2], but the mechanism by which mast cells alert and recruit neutrophils into the tissue is relatively unknown.

Mast cells are known to have broad biological function and regulate tissue inflammation in many disease settings including allergy, infection, autoimmunity, and cancer [3]. Interestingly, they have the potential to both initiate and inhibit inflammation during activation [4]. While mast cell–derived IL-10 has been shown to be necessary for inhibiting inflammation [5], the precise mechanisms through which mast cells initiate and promote tissue inflammation are not yet known. Our lab was the first to show that mast cells can express and upregulate the type 2 immune response–associated cytokine interleukin-33 (IL-33) upon IgE stimulation [6], but the physiological consequences for mast cell–derived IL-33 has remained unclear.

Similar to thymic stromal lymphopoietin (TSLP) and IL-25, IL-33 is understood to drive the initiation of type 2–associated immune responses [7]; IL-33 functions through its receptor, ST2, which is highly expressed on Th2 cells, mast cells, basophils, eosinophils, and type 2 cytokine–producing innate lymphoid cells (ILC2s). This is clinically relevant, as polymorphisms in both IL-33 and ST2 closely associate with asthma in human patients [8]. Mechanistically, recombinant IL-33 is sufficient to drive Th2 responses independent of IL-4 [9], and this Th2-skewing influence is likely due to effects of IL-33 on the priming potential of dendritic cells (DCs) since naïve T cells do not express ST2 [10]. Increased IL-33 is also commonly observed in tissues during active disease, such as in endobronchial biopsies from children with severe asthma [11], suggesting that IL-33 may not only participate in priming events but also in ongoing inflammatory effector responses. While the role of IL-33 in priming is well recognized, the role of IL-33 as an effector cytokine in the later phases of the immune response and during inflammation is less established.

One complicating factor in understanding the pathobiology of IL-33 is its reported expression by multiple cell types. IL-33 was originally identified as a constitutively expressed molecule in the nucleus of several structural cells, including fibroblasts, epithelial cells, and endothelial cells. Since then, several immune cells have also been shown to upregulate IL-33 upon stimulation: in addition to accumulating evidence demonstrating that mast cells express IL-33 upon activation [6, 12, 13], others have demonstrated inducible IL-33 expression in DCs [14, 15] and macrophages [16, 17] after activation. While much of the focus on IL-33 biology has been on its roles as both a transcriptional repressor and an “alarmin” released upon injury from the nucleus of cells with constitutive IL-33 expression [18], less is known about the roles of inducible IL-33 in immune cells. Studies by our group and others show that DC-derived IL-33 is sufficient to promote subsequent Th2 immunity in response to IgG immune complexes [14], and macrophage-derived IL-33 has shown a similar function to promote Th2 immunity during helminth infection [16]. In contrast to these innate roles, inducible expression of IL-33 by mast cells activated by antigen-specific IgE represents a role for IL-33 during events associated with adaptive immunity.

Passive cutaneous anaphylaxis (PCA) is an experimental animal model of allergic inflammation that features neutrophil-mediated inflammation, similar to what is observed in the late-phase reactions in human disease. Moreover, PCA is a passive-priming model that allows us to bypass the effect of IL-33 on priming and focus instead on determining whether it has any effector function during inflammation. In this mouse model, local cutaneous IgE administration is followed by systemic antigen exposure that elicits local antigen-driven tissue inflammation (ADTI) dominated by neutrophils. Previous studies from our group and others have unequivocally shown that ADTI after PCA is not only highly mast cell dependent [2] but also dependent on the IL-33 receptor, ST2 [6]. While activated neutrophils reportedly express ST2 [19], their weak chemotactic activity to IL-33 [20] precludes a direct role for ST2 in neutrophil infiltration during ADTI. Thus, how mast cells coordinate this neutrophil-mediated inflammation and how ST2 regulates this type of inflammation remains unexplained. Here, we establish a pathway through which mast cell–derived IL-33 indirectly initiates ADTI via activation of basophils. We recently reported a unique response profile in IL-33-activated basophils that was distinct from their “classic” IgE activation signature [21] in which they express neutrophil chemoattractants, such as CXCL1; here, we demonstrate that this process regulates late-phase neutrophil inflammation in the skin. Overall, our data demonstrate a functional effector role for mast cell–derived IL-33 in which it functions to initiate a molecular and cellular cascade that leads to the neutrophilic inflammation observed after exposure to allergens.

## Results

### ADTI responses require IL-33 and ST2

**Fig 1:**
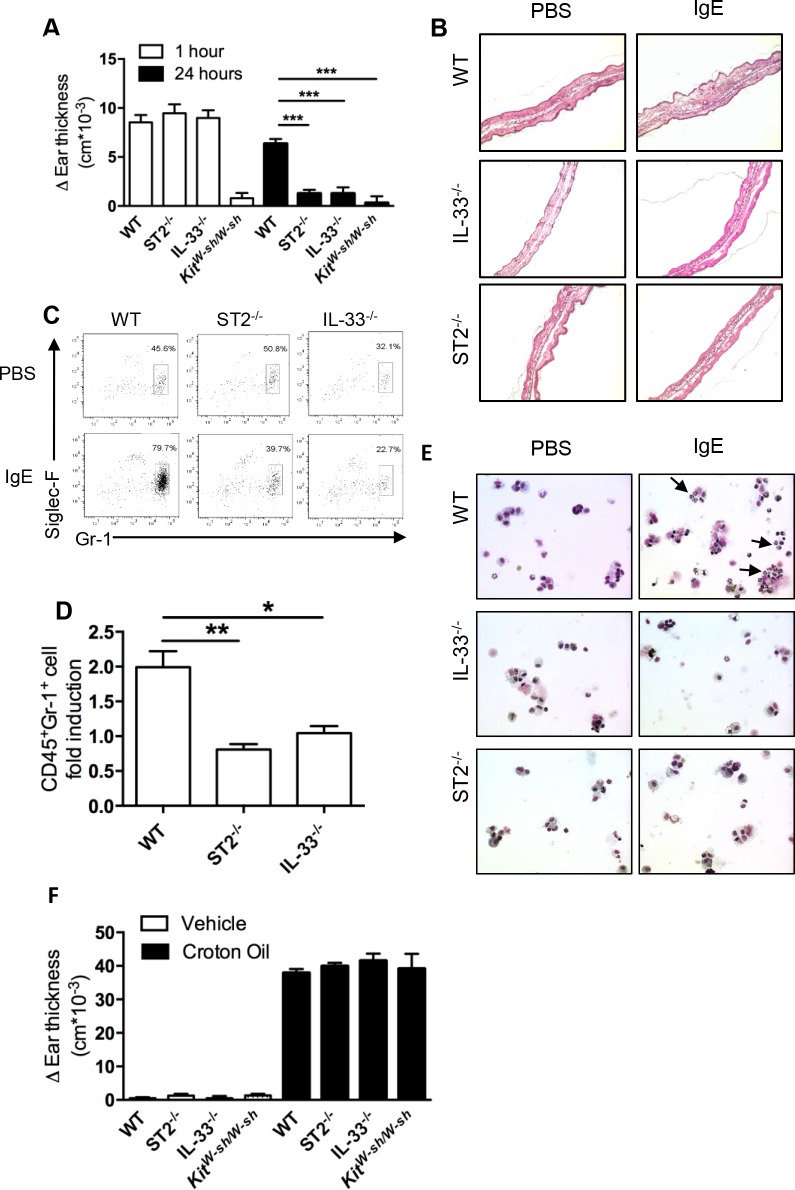
IL-33 and ST2 are necessary for ADTI. C57BL/6, *St2*^*–/–*^, or *Il33*^*–/–*^mice were used in the PCA model. Mice were intradermally injected with DNP-IgE followed by retroorbital challenge with DNP-HSA. (A) Ear thickness was measured at 0, 1, and 24 hours after antigen challenge. (B) Inflammatory cell infiltration was analyzed 24 hours after challenge by histological staining by H&E. (C, D) Flow cytometry flow plots and analysis of CD45^+^Gr-1^high^Siglec-F^low^ neutrophils and (E) cytospun BAL cells stained with Diff-quick. Arrows illustrate neutrophils. *P ≤ 0.05, **P ≤ 0.01 and ***P ≤ 0.001 (one-way ANOVA). (F) Ear thickness was measured 24 hours after application of croton oil. Data are from at least 3 independent experiments, and the mean ± SEM from n = 10–18 mice per group in (A) and n = 3–7 mice per group in (D) and (F) are displayed. Data in B, C, and E are representative of at least 3 independent experiments with similar results.

We previously demonstrated that IL-33 was elevated several hours into the time course of the mast cell–dependent PCA model and that ST2 was required for the ADTI response [6]. Since ST2 deficiency might also affect responses to stimuli other than IL-33 (e.g., some toll-like receptors [TLRs]) [22], we first sought to determine if endogenous IL-33 was the required signal for ST2 in ADTI. As predicted, both *St2*^*–/–*^and *Il33*^*–/–*^mice exhibited a normal response 1 hour after antigen challenge but failed to develop ADTI responses at 24 hours (Fig 1A). In contrast, mast cell–deficient mice (*Kit*^*W-sh/W-sh*^ *)* exhibited impaired responses at both the early and late time points (Fig 1A). While WT mice demonstrated the presence of tissue inflammation at this 24-hour time point (Fig 1B), this was noticeably absent in *St2*^*–/–*^and *Il33*^*–/–*^mice. Characteristic of this neutrophil-dominated ADTI response, the inflammatory cells were identified as CD45^+^Gr-1^high^Siglec-F^low^ neutrophils with polymorphic nuclei (Fig 1C, 1D and 1E) and were significantly reduced in both *St2*^*–/–*^and *Il33*^*–/–*^mice (Fig 1D). These observations suggest that both IL-33 and ST2 are necessary to support local, neutrophil-mediated tissue inflammation upon mast cell activation. Importantly, both *St2*^*–/–*^and *Il33*^*–/–*^mice remained capable of eliciting local skin inflammation in response to the non-specific, mast cell–independent irritant croton oil (Fig 1F).

### Mast cell–derived IL-33 is necessary and sufficient to elicit ADTI

**Fig 2:**
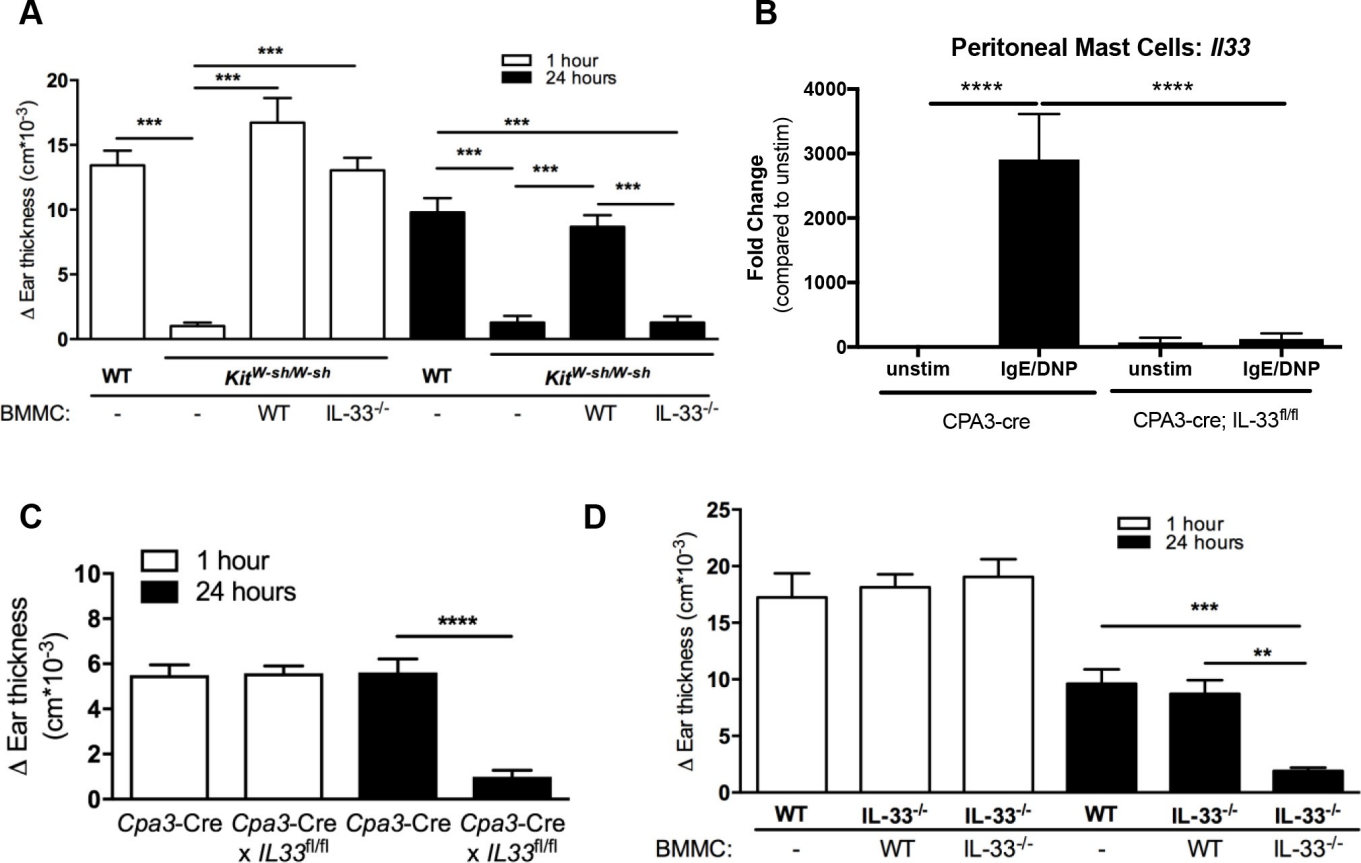
Mast cell-derived IL-33 is necessary and sufficient to elicit ADTI. (A) Ear skin of *Kit*^*W-sh/W-sh*^ mice was reconstituted after 8 weeks by intradermal injection of WT or *Il33*^*–/–*^BMMCs, and mice were rested for 1 week. Mice then underwent the PCA model as described in the methods. Ear thickness was measured at 0, 1, and 24 hours after challenge. (B) *Il33* mRNA was analyzed by real-time RT-PCR from peritoneal-cell derived mast cells harvested from *Cpa3-cre* and *Cpa3-Cre*×*Il33*^*fl/fl-IRES-eGFP*^ mice and stimulated with DNP-IgE/DNP-HSA for 4 hours. (C) *Cpa3-cre* and *Cpa3-Cre*×*Il33*^*fl/fl*^ mice underwent the PCA model as described in the methods and ear thickness was measured at 0, 1, and 24 hours after challenge. (D) Ear skin from WT or *Il33*^*–/–*^mice were intradermally injected with WT or *Il33*^*–/–*^BMMCs and rested for 1 week. Mice then underwent the PCA model as described in the methods. Ear thickness was measured at 0, 1 and 24 hours after challenge. **P ≤ 0.01, ***P ≤ 0.001 and ****P ≤ 0.0001 (one-way ANOVA). Data are from at least 2 independent experiments, and the mean ± SEM of n = 4–12 mice per group in (A), n = 5–6 mice per group in (B), n = 22–27 mice per group in (C) and n = 6–7 mice per group in (D) are displayed.

Although our data supported that ST2 and IL-33 were required for ADTI, the cellular source of IL-33 remained unknown. Since we previously showed that activated mast cells could be a source of IL-33 [6], we hypothesized that mast cells were the critical source of IL-33 after encountering the IgE-crosslinking antigen in our PCA model. A potential confounding issue is that mast cells can also *respond* to IL-33 through ST2 to elicit skin inflammation [23]. To avoid this issue and to discriminate between mast cell–derived IL-33 and any potential other sources that might support paracrine mast cell activation, we reconstituted mast cell–deficient *Kit*^*W-sh/W-sh*^ mice with WT or *Il33*^*–/–*^bone marrow–derived mast cells (BMMCs). We found that the absence of mast cells in unreconstituted *Kit*^*W-sh/W-sh*^ mice completely ablated both the early edematous response at 1 hour and ADTI at 24 hours (Fig 2A), confirming the critical role of mast cells in this model. While reconstitution with WT BMMCs restored both phases, reconstitution with *Il33*^*–/–*^BMMCs restored the early response but failed to restore ADTI (Fig 2A), indicating that mast cell–derived IL-33 was required for the ADTI response. To address the requirement of mast cell–derived IL-33 in another way, we also took advantage of available mice in which Cre recombinase is under the control of the mast cell–specific carboxypeptidase A3 (*Cpa3-Cre* mice) and crossed them to a floxed IL-33 (*Il33*^*fl/fl-IRES-eGFP*^) mice we previously generated [24] and with whom we had established the expression of IL-33 again by activated mast cells through the eGFP reporter signal both in vitro and in the skin of mice undergoing the ADTI model (S1 Fig). Initially, we verified that peritoneal mast cells from the *Cpa3-Cre*×*Il33*^*fl/fl-IRES-eGFP*^ mice were unable to express the *Il33* gene in response to IgE crosslinking––an established potent inducer of *Il33* mRNA and protein [6] (Fig 2B). As predicted by the mast cell reconstitution experiment, we observed complete ablation of the ADTI response at 24 hours after mast cell–specific deletion of IL-33, further confirming that mast cell–derived IL-33 was required for the ADTI response (Fig 2C).

To next test whether mast cell–derived IL-33 was sufficient to promote ADTI, WT or *Il33*^*–/–*^BMMCs were injected intradermally into the ear tissue of WT or *Il33*^*–/–*^mice 7 days prior to IgE priming (WT mice received WT BMMCs in order to control for potential changes in mast cell frequency). In contrast to the responses seen in Fig 1A, *Il33*^*–/–*^mice receiving WT, but not *Il33*^*–/–*^, BMMCs elicited a robust ADTI response (Fig 2D). Taken together, these results indicate that mast cell–derived IL-33 is not only necessary but also sufficient for initiating the cascade that allows late-phase inflammation to proceed during IgE-mediated responses to antigen.

### ST2-expressing basophils are required for ADTI responses

**Fig 3:**
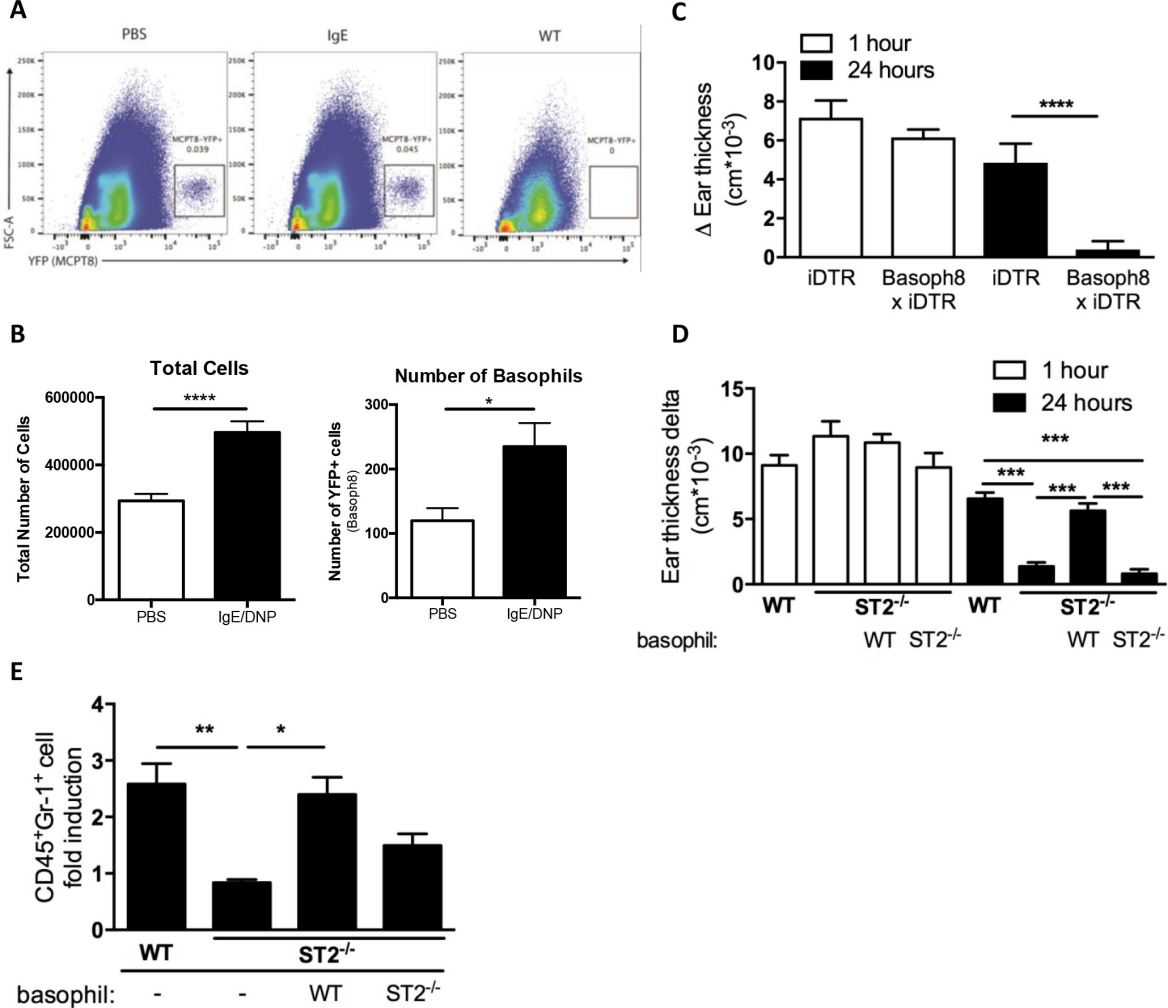
ST2-expressing basophils are required for ADTI responses. (A) Representative gating for basophils (MCPT8-YFP+) and (B) quantification of total cells and basophils in PBS and DNP-IgE/DNP-HSA treated ears 2 hours after challenge. (C) WT x iDTR (control) and Basoph8 x iDTR mice received 3 consecutive doses of DT (1 μg) for basophil depletion. Mice then underwent the PCA model as described in the methods. Ear thickness was measured at 0, 1 and 24 hours. (D) C57BL/6 WT and *St2*^*–/–*^mice received WT or *St2*^*–/–*^BMBs, and the PCA model was performed 1 hour later. Ear thickness (D) was measured at 0, 1, and 24 hours after challenge, and ear neutrophil population (CD45^+^Gr-1^high^Siglec-F^low^) was analyzed by flow cytometry (E). *P ≤ 0.05, **P ≤ 0.01, ***P ≤ 0.001 and ****P ≤ 0.0001 (one-way ANOVA). Data from (A, B) are from at least 2 independent experiments. Data from (C, D) are from at least 4 independent experiments, and the mean ± SEM from n = 11 (B), n = 9–17 (C), n = 12–17 (D) and n = 7–15 mice per group (E) are displayed.

Although we established that mast cells produced the IL-33 required for the ADTI response, the identity of the ST2-bearing cell necessary to carry forward the IL-33 signal and recruit neutrophils in ADTI remained unknown. The relatively low ST2 expression on resting neutrophils and the weak chemotactic ability of activated neutrophils to IL-33 [19, 20] suggested that a direct effect of mast cell–derived IL-33 on neutrophil migration was unlikely and that cells with constitutive expression of ST2 might be involved instead in our passive priming model. Since previous work showed that basophils expressed ST2 [25] and were required for initiating skin inflammation in a chronic allergic skin model that included a strong neutrophil component [26], we hypothesized that basophils might be sensing mast cell–derived IL-33. Since basophils are known to circulate in the blood and be recruited to local tissues only upon inflammation, we initially assessed whether basophils entered into the local environment of the ear by tracking YFP-positive basophils in *Mcpt8*^*tm1(cre)Lky*^/J (Basoph8) mice, which have a built-in YFP signal attached to the *Mcpt8* gene [27]. We observed a significant increase in the frequency (Fig 3A) and total number (Fig 3B) of basophils in the ear 2 hours after challenge in the PCA model, indicating that basophils were recruited locally to the ear tissue upon challenge.

To formally test whether basophils were required for eliciting the ADTI response, we made use of Basoph8 mice crossed to iDTR mice (Basoph8×iDTR) in which basophils could be selectively depleted upon diphtheria toxin (DT) treatment via Cre-inducible DTR expression on basophils. In the PCA model, we found that basophil-depleted mice were unable to mount the late-phase ear swelling response at 24 hours that normally occurred in WT control mice (Fig 3C), suggesting that basophils were required for the ADTI response. In support of these findings, we also depleted basophils using an anti-mFcεR1 antibody (MAR-1) [28] (S2 Fig) and observed a significantly diminished response to antigen at both 1 hour and at 24 hours. Furthermore, repletion of bone marrow–derived basophils (BMBs) from *St2*^*+/–*^, but not littermate *St2*^*–/–*^, mice into MAR-1–depleted recipients was sufficient to restore the ADTI response (S1 Fig, panel C), indicating that ST2 signaling on basophils was important to their role in ADTI.

To test whether ST2 signaling on basophils was required for their role in ADTI and to rule out any effect of anti-mFcεR1 antibody on mast cell functions on the diminished edematous response (although no decrease in the numbers of skin mast cells was noted), we next utilized the basophil repletion approach in *St2*^*–/–*^recipient mice that lacked ADTI reactivity, as shown in Fig 1A. WT, but not *St2*^*–/–*^, BMBs fully restored ADTI to *St2*^*–/–*^mice (Fig 3D), and these responses correlated well with the percentage of neutrophils in the tissue of the respective mice (Fig 3E). Thus, rather than directly initiating neutrophilic inflammation, mast cell–derived IL-33 requires ST2-bearing basophil intermediates to recruit neutrophils.

### ST2-driven stimulation of basophils promotes a unique inflammatory profile

**Fig 4:**
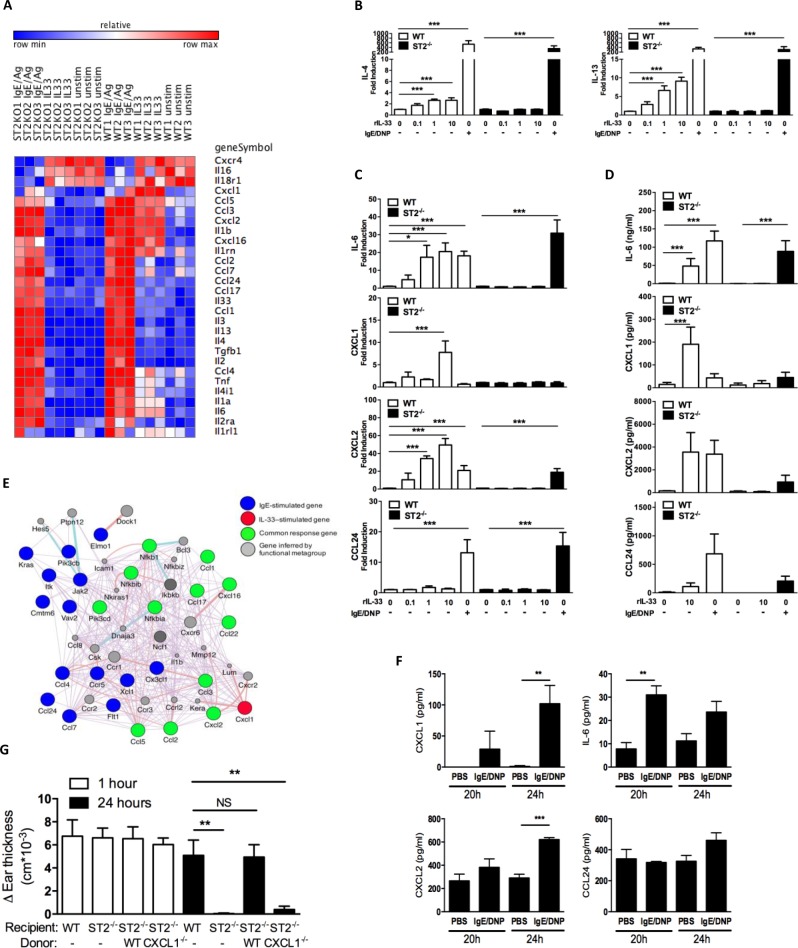
ST2-driven stimulation of basophils promotes a unique inflammatory profile in which basophil-derived CXCL1 is functionally important in their involvement in ADTI. (A–C) BMBs from C57BL/6J mice were activated with IgE/DNP or different doses of mIL-33. Gene expression 4 hours after activation was analyzed by microarray (A) and real time RT-PCR (B, C). Protein production 24 hours after activation was determined by ELISA (D). (E) Network diagram generated from the microarray comparison of activated BMBs in (A) showing select genes altered by IgE-stimulation, IL-33-stimulation and shared by both stimuli. (F) Ear tissues from C57BL/6J mice undergoing the PCA model were collected at 20 and 24 hours after challenge and homogenized. IL-6, CXCL1, CXCL2 and CCL24 expression was determined by ELISA and normalized to total protein concentration. Data are from 3 individual mice at each time point (mean ± SEM). (G) WT or *St2*^*–/–*^underwent the PCA model. Some mice underwent repletion with WT or *Cxcl1*^*–/–*^BMBs 1 hour before challenge. Ear thickness was measured at 0, 1 and 24 hours after challenge. *P ≤ 0.05, **P ≤ 0.01, and ***P ≤ 0.001 (one-way ANOVA). Data are from at least 2 independent experiments, and mean ± SEM for n = 3–11 per group (B, C) n = 4–9 per group (D), n = 3 per group (F), and n = 6–10 per group (G) are displayed.

How, then, do IL-33–stimulated basophils recruit neutrophils into the tissue? Basophil responses are heterogeneous depending on the type of stimuli; for example, basophil output responses observed after TSLP-driven basophil activation differ from IgE/antigen–mediated activation [29]. We therefore asked what type of responses IL-33 activation would elicit and whether these responses differ from those of IgE/antigen–activated basophils. We initially used microarray analysis on BMBs *in vitro* as an unbiased approach to address these questions and observed that IL-33 stimulation led to a unique expression profile of inflammatory mediators that differed from that of IgE/antigen stimulation (Fig 4A). While IgE/antigen supported a robust profile of cytokine and chemokine responses, IL-33 activation was surprisingly characterized by the presence of inflammatory-type chemokines and cytokines as well as the absence of Th2-associated signatures (e.g., IL-4, IL-13, CCL11, CCL17, and CCL24). Since this was in contrast to some previous studies concluding that IL-33 stimulation induced basophils to produce type 2 cytokines such as IL-4 and IL-13 [30–32] [33, 34], we used real-time RT-PCR to validate our findings. Although we were able to detect some small dose-dependent fold-change increases in both IL-4 and IL-13 in BMBs after IL-33 stimulation, these changes were much less than the IL-4 (~200×) and IL-13 (~40×) expression that occurred upon stimulation with IgE/Ag (Fig 4B), a finding consistent with work from another group on primary human basophils [35]. Among the genes we did identify as being associated primarily with IL-33, IL-33 was as good or better than IgE/antigen activation for inducing mRNA (Fig 4C) and protein (Fig 4D) expression of the neutrophil-attracting mediators IL-6, CXCL1, and CXCL2 but not the Type 2–associated chemokine CCL24 (eotaxin-2), an eosinophilic-attracting chemokine selectively increased under IgE/antigen–stimulated conditions. Importantly, these changes did not occur in *St2*^*–/–*^basophils, assuring us that these changes were IL-33 dependent (Fig 4A). These data suggest that IL-33 activation promotes a unique and highly selective response profile in basophils attuned to facilitate inflammatory responses that differs dramatically from the IgE-driven activation profile that associates instead with Type 2 immunity.

As another method to identify the key genes specifically regulated by IL-33 based on our microarray data, we constructed a focused network diagram on the chemokine signature alone. This network map elegantly illustrates the distinct IL-33–mediated basophil response signature featuring neutrophil-attracting molecules and importantly reveals that the CXCL1 chemokine in particular is upregulated only in response to IL-33 stimulation (Fig 4E).

### The IL-33–activated basophil response signature is evident during ADTI responses in vivo, and basophil-derived CXCL1 is required for the hallmark neutrophil infiltration in ADTI

Given the unique response profile we observed in *in vitro* activation of BMBs by IL-33, we next asked whether this signature was activated during the course of the IL-33–dependent ADTI response *in vivo* in which we previously showed that IL-33 levels were elevated [6]. Indeed, skin tissue homogenates taken from the site of IgE injection exhibited significantly elevated IL-6 protein levels at 20 hours after antigen challenge as well as significantly elevated CXCL1 and CXCL2 at 24 hours compared to PBS-injected skin from the same animals; in contrast, antigen challenge did not alter CCL24 levels away from the PBS-control skin at either time point (Fig 4F). These data echo the unique inflammatory basophil signature from the IL-33–activated BMBs *in vitro* and further suggest that ST2-bearing basophils may play an intermediary role between mast cell–derived IL-33 and neutrophil infiltration during the course of ADTI *in vivo*.

To test whether the neutrophil-attracting response elicited by IL-33 activation of basophils was required for eliciting the ADTI response, we capitalized on the information gleaned from the network map identifying CXCL1 as the unique basophil-derived factor upregulated upon IL-33/ST2–mediated stimulation and adoptively transferred WT or *Cxcl1*^*–/–*^BMBs into *St2*^*–/–*^recipient mice before performing PCA. As shown in Fig 4G, repletion of *St2*^*–/–*^mice with WT, but not *Cxcl1*^*–/–*^, BMBs fully restored the lack of ADTI responsiveness in unmanipulated *St2*^*–/–*^mice at the 24-hour time point. These data support the conclusion that basophils activated by mast cell–derived IL-33 play a key role in driving the characteristic neutrophil infiltration of ADTI through their production of CXCL1.

## Discussion

Significant attention has been paid to the idea that IL-33 is a tissue-derived, constitutively expressed cytokine that resides in the nucleus of structural cells and acts as an alarmin when released after cellular damage [36]. The concept that other cells are able to produce IL-33 upon stimulation (inducible IL-33), including CD45^+^ hematopoietic cells, may also be a physiologically relevant source of IL-33 has been challenged based on the magnitude of differences in IL-33 expression levels observed in these cells when compared to higher IL-33–expressing structural cells [36]. Genome-wide association studies (GWAS) strongly link several single nucleotide polymorphisms (SNPs) in both IL-33 and ST2 to asthma [37]; interestingly, many of the most strongly correlated SNPs in IL-33 occur in the 5´ region of the gene important for inducing gene transcription, implying that the cells capable producing IL-33 upon stimulation rather than those with constitutive IL-33 expression play roles in asthma disease pathogenesis. The data presented herein provides evidence to support the conclusion that inducible IL-33 from hematopoietic cells does play functional roles in allergic disease pathogenesis and offer a deeper understanding for how such sources of IL-33 influence inflammation. We demonstrate that mast cells are a necessary and sufficient cellular source of IL-33 during the IgE-mediated late-phase allergic inflammation characterized by neutrophilic infiltration. Additionally, while the importance of IL-33 in allergic disease has thus far been attributed to its effect on priming type 2 immunity, our findings establish a mechanistic link between IL-33 and neutrophilic inflammation during allergic inflammation: rather than a direct effect on neutrophils, our data support that mast cell–derived IL-33 functions by inducing a basophil intermediary to secrete neutrophil-attracting chemokines.

To date, the physiologically relevant sources of IL-33 have been difficult to determine; moreover, the precise roles of tissue-derived versus hematopoietic cell–derived IL-33 have remained unclear. In a clinical study, rhinovirus infection increased IL-33 in the airway of asthma patients [38]; even though this particular study also showed that rhinovirus increased IL-33 from bronchial epithelial cells in an *ex vivo* experiment, the true IL-33–producing cells *in vivo* was not identified. Another study showed that asthma severity in children correlated with IL-33 expression in submucosal regions––a region in which many hematopoietic-derived cells reside––rather than in the epithelium or smooth muscle [11]. Apart from these human studies, animal studies have also attempted to tease apart the importance of the various IL-33–producing cells. A study using LacZ-based reporter mice concluded that IL-33 was predominantly present in epithelial barriers and other structural tissues [39]. In contrast, a citrine reporter strain showed expression only in the type-2 pneumocytes of the lung and observed IL-33 expression in macrophages, DCs, eosinophils, and B cells upon allergen-driven inflammation [40]. In collaboration with the Herbert group, we showed that macrophages and inflammatory DCs were implicated in Th2 responses following hookworm infection and that DC-derived IL-33 was sufficient to support Th2 skewing *in vitro* [16]. Work by the Sperling group supported a similar role for DC-derived IL-33 in an immune complex–mediated model of Th2 priming and asthma [14, 15]. Another study in the house dust mite–induced airway inflammation model showed that monocytes recruited to the lung were a source of IL-33 [41]. Thus, the concept that non-epithelial cells are important contributors of IL-33 has been established but remains underappreciated. Here, our data argue that inducible IL-33 exclusively from hematopoietic-derived mast cells indeed plays a functional role in a model of neutrophil-mediated allergic inflammation.

As mentioned in the Introduction, IL-33 immunologic function has been strongly linked to Th2 priming events [7]; indeed, we previously described that DC-derived IL-33 was sufficient to support Th2 priming [14], but the experimental approach used in that study did not rule out the potential for other IL-33 sources. We therefore eliminated this possibility in the present study by transferring WT mast cells into *Il33*^*–/–*^mice and showing that these IL-33–containing mast cells were sufficient to elicit tissue inflammation upon mast cell activation. Importantly, this IL-33 production was, by its very nature, functionally distinct from the influence of IL-33 over priming since the presence of antigen-specific IgE to stimulate mast cells required an established priming response. Thus, in addition to its role in priming, our work establishes that IL-33 also plays a novel role in adaptive immune responses mediated by antigen-specific IgE.

The mechanisms through which mast cells regulate tissue inflammation after being stimulated have remained elusive. The strong dependency of the PCA model on mast cells––evident by the complete absence of either the initial or later-phase ADTI responses and recovery of these responses upon mast cell reconstitution––allows interrogation of such mechanisms without the complication of mediators that exert effects on immune priming, which would certainly need to be taken into consideration when studying IL-33 [7]. Early studies suggest that mast cell–derived TNF may be a possible mechanism regulating the ensuing tissue inflammation [2], and this has been supported in other studies [42]. Interestingly, TNF has been shown to enhance IL-33 expression in keratinocytes [43], but whether this also applies to mast cells remains unclear. Recently, neutrophil recruitment in response to recombinant IL-33 has been shown to be mast cell dependent [42], placing mast cells upstream of IL-33 and contrasting with our findings. IL-33 is undoubtedly a potent inducer of mast cell cytokines, including those relating to neutrophil responses [6, 42, 44, 45]. However, while IgE/Ag–driven mast cell activation is sufficient to induce IL-33 expression, we have previously shown that IgE/Ag–driven activation associates with both downregulated ST2 mRNA expression and increased ST2 shedding from the cell surface [6], suggesting that mast cells driven to express IL-33 are also likely to be refractory to an autocrine mechanism of activation. Since our data firmly establishes the importance of basophils as an intermediate cell during the ADTI response, a direct influence of mast cells on neutrophils during IgE-mediated activation also seems to be a highly unlikely mechanism.

Some previous studies conclude that IL-33–stimulated basophils produce type 2 cytokines, but this finding is controversial with reports both for and against IL-4 expression [30–35]. In line with our reported studies on the activation profiles between IgE/Ag–stimulated vs IL-33–stimulated basophils [21], we observed that IL-33 mainly affected inflammatory-associated cytokine and chemokines while IgE/Ag activation drove a relatively stronger type 2–associated cytokine response. Another interesting finding from our activation profile comparison of basophil activation was that IgE/Ag stimulation induced IL-33 expression from basophils while IL-33 did not, similar to what we previously showed for mast cell activation [6], implying perhaps that basophils might also be a source of IL-33 during IgE-associated responses.

Focused network map analysis using the data generated from our microarray analysis identified the neutrophil-attracting chemokine CXCL1 as uniquely regulated by IL-33 activation of basophils. This finding is consistent with previous *in vitro* studies on human basophils showing that peripheral blood–derived basophils are a direct target of IL-33 and that IL-33 promotes secretion of IL-8––the human homologue of CXCL1––when in the presence of IL-3 [31, 46], similar to the conditions our *in vitro* BMBs experienced. Additionally, the diminished ADTI responses we observe upon transfer of *Cxcl1*^*–/–*^BMBs compared to WT BMBs into *St2*^*–/–*^recipient mice supports not only that basophil-derived CXCL1 is a critical event during the initiation of tissue inflammation by mast cell activation but also that this is highly dependent on IL-33. This can provide one mechanistic explanation for the observations in the literature showing that basophils are at the site of edema and may be involved in the response [47–49]. Within the wheals that developed after initiation of an autologous serum skin test (ASST) in human patients with chronic idiopathic urticaria (CIU), a significant increase in basophils was observed at 30 min, preceding the peak of neutrophil infiltration at 60 min [47]; another study also observed increased basophils at the site of urticaria in human patients [48]. Here, we show a significant increase in basophils in the ear after challenge, similar to what has previously been observed in other allergic models [50], and that these basophils are absolutely required for the late-phase inflammation in both the Basoph8×ROSA26iDTR mouse and MAR-1 depletion experiments. Thus, basophils are poised shortly after antigen triggering to play a critical functional role in recruiting neutrophils to the site of inflammation. In further support of what we show here, another group has shown that basophil depletion reduced neutrophil infiltration into the skin in an IgE-mediated cutaneous reverse passive Arthus model in mice [49].

Indeed, our data fully support the basophil being a key responder cell type in IL-33–mediated responses since repletion of WT basophils fully restores responses in *St2*^*–/–*^mice. This finding raises questions regarding the contribution of other ST2-bearing cells, such as ILC2s, in this type of inflammation. Although ILC2s have been shown to be a critical cell in atopic dermatitis–like inflammation in a skin-specific IL-33–transgenic mouse (IL-33 under the control of the keratin 14 promoter) [51], this effect may likely be from influences over immunological priming rather than on the inflammation itself since these mice acquire a spontaneous dermatitis as well as exhibit high levels of both IgE and Type 2 cytokines. Mast cells and ILC2s may interact with each other during allergic inflammation––dermal ILC2s have been described to co-localize with mast cells [52]––but some previous studies suggest mechanisms other than IL-33 for their cross-talk. For example, mast cell–derived PGD_2_, but not IL-33, can potently induce ILC2 migration via CRTH2 expression [53], and ILC2-derived IL-13 may play a role in suppressing mast cell activation [52].

In conclusion, the proposed mechanism described by our work provides an in-depth understanding of the biology of IL-33 and establishes that mast cells are a physiologically important source of this cytokine during the adaptive immune response, defined here by antigen crosslinking of antigen-specific IgE. Surprisingly, the necessity for ST2-bearing basophils as the responder cell to mast cell–derived IL-33 places them in a unique mechanistic pathway to orchestrate a cascade of events leading to neutrophilic inflammation that is well beyond the established roles these cells are known to play in immunity. The significance of our findings might reach well beyond allergy, especially since mast cells also participate in a variety of inflammatory settings that involve neutrophilic inflammation (e.g., tumorigenesis [54], autoimmunity [55], infection [56, 57]) and IL-33 is likely involved in several other inflammatory diseases (e.g., rheumatoid arthritis [19], ankylosing spondylitis [20], psoriasis [23], cystic fibrosis [58], antiviral Th1-mediated immunity [59]).

## Experimental procedures

### Animals

C57BL/6J, *Kit*^*W-sh/W-sh*^, *Cpa3-Cre*, and *Il6*^*–/–*^, Basoph8 (Mcpt8^***tm1(cre)Lky***^**/**J**)**, and iDTR (Gt(ROSA)26Sor^*tm1(HBEGF)Awai*^/J) mice were obtained from the Jackson Laboratory (Bar Harbor, ME). *St2*^*–/–*^mice were previously described [60] and backcrossed 8 generations onto the C57BL/6J strain. *Il33*^*–/–*^mice (C57BL/6J background) were provided by Amgen Inc. Mice were maintained in specific pathogen-free conditions at Northwestern University Center for Comparative Medicine. All protocols were approved by the Northwestern University Animal Care and Use Committee. Isoflurane was used for anesthesia and euthanasia, as appropriate. Bone marrow from *Cxcl1*^*–/–*^mice was kindly provided by Dr. Samithamby Jeyaseelan at Louisiana State University (Baton Rouge, LA) (C57BL/6J background) and were previously described [61].

### Il33^fl/fl-IRES-eGFP^ generation

*Il33*^*fl/fl-IRES-eGFP*^ were generated by the InGenious Targeting Laboratory (Ronkonkoma, NY). The targeting vector was designed to contain LoxP sites flanking exons 5 and 7, a FRT-containing neomycin cassette for antibiotic selection, and an IRES-GFP cassette within exon 8 (S1 Fig, panel A). The linearized vector was transfected into C57BL/6 embryonic stem (ES) cells by electroporation. After G418 antibiotic selection, the recombinant ES clones were identified by PCR analysis. ES cells were then injected into C57BL/6 blastocysts to generate chimeras. The Neo cassette was then deleted in the vector-containing offspring by crossing them to mice that express the Flp recombinase in the testis, and the F1 generation were considered to be the floxed IL-33 mice. Floxed IL-33 were identified using the following primers: ITLE3, 5′-GTCCAAGTCTGCTTCAGTTTACCC -3′ and SDL2, 5′-AGTACCATAGCTGATACCAGGGTG-3′ in which the WT allele gives a 593 bp band and the vector-containing allele gives a 541 bp band. To ensure that activated mast cells from our floxed IL-33 mice (the IL-33 gene is tagged with eGFP) expressed IL-33 and confirm our original published finding that activated mast cells were capable of expressing IL-33 [6], we tested the ability of these mast cells to express IL-33 both *in vitro* and *in vivo*. IgE activation alone induced eGFP-tagged IL-33 expression in 5% of BMMCs *in vitro* (S1 Fig, panel B) as well as in the PCA model *in vivo*, where the frequency of GFP^+^ mast cells significantly increased in the ear 16 hours after IgE activation (S1 Fig, panel C). We confirmed that this GFP^+^ population represented the *Il33*-expressing population by sorting the GFP^+^ from the GFP^−^BMMCs grown from the floxed IL-33 mice and performing real-time RT-PCR for *Il33* (S1 Fig, panel D).

### Generation of mice with mast cell–specific deletion of Il33

*Il33*^*fl/fl-IRES-eGFP*^ were bred with *Cpa3-Cre* mice to generate *Cpa3-Cre*×*Il33*^*fl/fl-IRES-eGFP*^ mice in whom IL-33 is deleted specifically from mast cells.

### Passive cutaneous anaphylaxis

Mice were intradermally sensitized with 100 μg dinitrophenyl-specific IgE (DNP-IgE, Sigma) in one ear and PBS as the control in the other ear. After 16–18 hours, mice were retroorbitally challenged with 100 μg DNP-coupled albumin (DNP-HSA, Sigma). Ear thickness was measured at 0, 1, and 24 hours after challenge, as previously described [6]. Ears were harvested 24 hours after challenge. Cellular infiltration was analyzed by histology of tissue sections and cytospun cells as well as by flow cytometry. Cytokine expression was determined by ELISA; obtained values were normalized to total protein determined by Pierce BCA protein assay kit (Thermo Scientific). In some experiments, donor basophils were retroorbitally transferred into recipients 1 hour before challenge. Differences in ear thickness were determined by subtracting the baseline 0-hour measurement from the measurement at 1 or 24 hours after challenge.

### Croton oil-induced skin inflammation

For each mouse, 20 μL of acetone (vehicle) was applied on both sides of one ear and 20 μL of 5% croton oil (Sigma) on both sides of the other ear. Ear thickness was measured at 0 and 24 hours. Differences in ear thickness were determined by subtracting the baseline 0-hour measurement from the measurement taken at 24 hours after challenge.

### Peritoneal cell-derived mast cells and real-time RT-PCR

As previously described, peritoneal lavage was performed on mice using 8–10 mL of cold lavage fluid (10% FBS and 1mM EDTA[62]. Cells were centrifuged and resuspended in 5 mL media (RPMI 1640 media [Corning] containing 10% FBS [Atlanta Biologicals], 2.5% HEPES [Sigma], 1% sodium pyruvate [Sigma], 1% non-essential amino acids [Sigma], 1% penicillin/streptomycin [Corning], and 0.05 mM β-mercaptoethanol [Sigma]) with 10 ng/mL recombinant mIL-3 and 30 ng/mL recombinant mSCF. On Day 2 and 5, media was removed without disturbing adherent cells, and fresh media with mIL-3 and mSCF was added. On Day 12–14, suspension cells were harvested, and purity was checked by flow cytometry (double-positive for APC–anti-mCD117 [2B8, BD Biosciences] and PE–anti-mFcεRI [MAR-1, eBioscience]) before use. Cellular mRNA was extracted using the RNeasy kit (Qiagen), and cDNA was generated with the qScript cDNA synthesis kit (Quanta BioSciences). Gene expression was determined by real-time PCR using an ABI 7500 thermal cycler (Applied Biosystems) and specific TaqMan probes (Applied Biosystems) for each gene of interest. β-actin expression was used as an internal control, and changes in the threshold cycle values were determined.

### Histology

Ear tissues were harvested 24 hours after challenge and fixed in formalin in the lab. Embedding in paraffin, sectioning, and staining with H&E were performed by the Mouse Histology and Phenotyping Laboratory at Northwestern University.

### Ear digestion

Mouse ears were harvested and split into dorsal and ventral parts. Tissues were cut into small pieces and incubated in HBSS containing 1 mg/mL collagenase D (Sigma) and 0.1 mg/mL DNase (Roche) at 37°C for 2 hours followed by filtering through 70 μM strainer (BD Biosciences) and washing with PBS containing 1% FBS.

### Flow cytometry

Single-cell suspensions from ear digestion were adjusted to a density of 1 × 10^6^ cells/mL, and non-specific binding was blocked with anti-mCD16/CD31 (2.4G2, BD Biosciences) for 15 minutes at 4°C. Cells were then stained with pacific blue–anti-mCD45 (30-F11, BioLegend), PerCP-Cy5.5–anti-mGr-1 (RB6-8C5, BD Biosciences) and PE–anti-mSiglec-F (E50-2440, BD Biosciences) at 4°C for 30 minutes. Samples labeled with these flow cytometry antibodies were collected with an LSR II (BD Biosciences), and then data were analyzed with FlowJo software (BD Biosciences). Cells were gated as CD45^+^ singlets. Fold induction of neutrophil (CD45^+^Gr-1^high^Siglec-F^low^) percentage was compared between IgE-sensitized ear and its own PBS control ear.

### Cytospin

Cells (500,000) were spun at 400 rpm for 4 minutes onto the slides. After air drying, cells were stained with Wright–Giemsa stain (Kwik-Diff, Thermo Scientific).

### Bone marrow–derived mast cell (BMMC) generation and reconstitution

Bone marrow was obtained by flushing from mouse femurs and tibias using RPMI 1640 media (Corning) containing 10% FBS (Atlanta Biologicals), 2.5% HEPES (Sigma), 1% sodium pyruvate (Sigma), 1% non-essential amino acids (Sigma), 1% penicillin/streptomycin (Corning), and 0.05 mM β-mercaptoethanol (Sigma) (BMMC media). Cells were then cultured in BMMC media with 30 ng/mL recombinant mIL-3 (Miltenyi Biotec) for 4–5 weeks. Mast cell phenotype was determined by flow cytometry as cells staining double-positive for APC–anti-mCD117 (2B8, BD Biosciences) and PE–anti-mFcεRI (MAR-1, eBioscience). BMMCs with a >95% pure were used. For mast cell reconstitution, BMMCs were injected intradermally for 8 weeks in the experiments to determine necessity and for 1 week in the experiments to determine sufficiency.

### Basophil depletion by DTR

For basophil-specific depletion, Basoph8×iDTR or C57BL/6J×iDTR (control) mice were injected intraperitoneally for three consecutive days with 1 μg of diphtheria toxin (Sigma D0564) in 200 μL (0.1%BSA in PBS). On day 3, the mice were injected with IgE intradermally. On day 4, the mice were challenged retroorbitally, and ear thickness measurements were taken at 0, 1, and 24 hours.

### Basophil depletion by MAR-1 antibody

C57BL/6 mice were retroorbitally injected with 10 μg anti-mFcεRI antibody (MAR-1, eBioscience) in PBS for 3 consecutive days prior to IgE priming. The control group received PBS alone. The depletion efficacy was determined by flow cytometry staining with APC–anti-mCD49b (DX-5, BD Biosciences) and PE–anti-mFcεRI (MAR-1, eBioscience); FcεRI^+^DX-5^+^ basophils were almost completely eradicated from the spleen after MAR-1 treatment after the 3-day depletion (S2 Fig, panels A and B).

### Ear digestion and basophil enumeration

Ear tissue was harvested and separated into ventral and dorsal halves. Tissue was then placed in HBSS with 2 U/mL Liberase TM (Rocher) and minced with scissors. Homogenates were incubated for 30min at 37°C with constant agitation. Digestion was stopped with cold 10% fetal bovine serum and samples were strained through a 70 μm strainer. Total cell numbers were counted and then samples were stained for flow cytometry.

### Bone marrow-derived basophil (BMB) generation, repletion, and activation

Bone marrow was flushed from mouse femurs and tibias using BMMC media. Cells were cultured with BMMC media containing 30 ng/mL recombinant mIL-3 (Miltenyi Biotec). At day 9, suspended cells were first negatively depleted with mCD117 MicroBeads (Miltenyi Biotec) and then positively selected with mCD49b (DX-5) MicroBeads (Miltenyi Biotec) using the autoMACs instrument (Miltenyi Biotec). The purity of isolated BMBs was determined by flow cytometry; BMBs were identified by positively staining for APC–anti-mCD49b (HMα2, BD Biosciences) and FITC–anti-mFcεRI (MAR-1, eBioscience) but negatively staining for PE–anti-mCD117 (2B8, BioLegend). For basophil repletion, BMBs were retroorbitally transferred 1 hour before antigen challenge. BMBs were primed with 1 μg/mL DNP-IgE (Sigma) overnight followed by stimulating with 0.5 μg/mL DNP-HSA (Sigma) for 4 hours (RNA) and 24 hours (protein). BMBs were also activated with different doses of recombinant mIL-33 (R&D Systems).

### Microarray analysis and real-time RT-PCR

BMBs were activated with either IgE/DNP or 10 ng/ml mIL-33 (R&D Systems) for 4 hours. mRNA was extracted by RNeasy kit (Qiagen) and cDNA was generated by qScript cDNA synthesis kit (Quanta BioSciences). Microarray assessment was performed by the Northwestern University Genomics Core using llumina MouseWG-6 Beadchips. Heatmaps were generated using GENE-E software (Broad Institute, http://broadinstitute.org/cancer/software/GENE-E). Network diagrams were generated using the “FGNet” R package and the GeneTerm Linker algorithm (adjusted *P* value < 0.05; minimum support of 3). Visualization of these networks was performed with the “iGraph” R package and Cytoscape 3.2.1. Gene expression was also determined by real-time PCR using an ABI 7500 thermal cycler (Applied Biosystems) and specific TaqMan probes (Applied Biosystems) for each gene of interest. β-actin expression was used as an internal control, and changes in the threshold cycle values were determined.

### ELISA

IL-6, CXCL1, CXCL2, and CCL24 DuoSet ELISA kits were obtained from R&D Systems. The ELISA was performed according to the manufacturer’s instructions.

### Statistics

Statistics were performed on GraphPad Prism 6 software (GraphPad Software, La Jolla, CA) using two-tailed Student’s *t* test or one-way ANOVA to determine significance. *P < 0.05; **P < 0.01; ***P < 0.001.

## Supporting information

S1 Fig Construction and characterization of *Il33*^*fl/fl-IRES-eGFP*^ mice and cross to *Cpa3-Cre* mice. (A) Design and construction of the conditional *Il33*^*fl/fl-IRES-eGFP*^ knock-in targeting vector. (B) BMMCs were generated from *Il33*^*fl/fl-IRES-eGFP*^ and checked for purity as described in the methods. For IgE-mediated activation, BMMCs were coated *in vitro* with ovalbumin-specific IgE overnight and activated with ovalbumin (OVA) for 24 hours. Cells were harvested and analyzed by flow cytometry. IL33-GFP^+^ cells (% of live cells) is reported in the representative flow cytometry figures. (C) *Il33*^*fl/fl-IRES-eGFP*^ mice underwent the PCA model with DNP-IgE/DNP-HSA as described in the methods. Ears were harvested 16 hours after challenge and digested for flow cytometry. IL33-GFP^+^ cells (of live F4/80^–^CD44^+^CD117^+^ mast cells) were identified by flow cytometry and quantified. (D) BMMCs from the *Il33*^*fl/fl-IRES-eGFP*^ were activated as described in (B). GFP^−^and GFP^+^ cells were flow sorted and harvested for RNA. *Il33* mRNA levels were quantified by RT-PCR. *P ≤ 0.05 and **P ≤ 0.01 (one-way ANOVA). Data are from at least 2 independent experiments, and the mean ± SEM of n = 7 (C) and n = 3–5 mice per group are displayed.
(TIFF)

S2 Fig ST2-expressing basophils are required for ADTI responses by MAR-1 depletion. (A, B) C57BL/6J mice were retroorbitally injected with 10 μg anti-mFcεRI or PBS for 3 constitutive days. Representative flow data (A) and quantification (B) of spleen basophil population (CD49b^+^FcεRI^+^). Data is represented as mean ± SEM of n = 5 per group. ***P ≤ 0.001 (two-tailed Student’s *t* test). (C) C57BL/6J mice received PBS or 10 μg anti-mFcεRI (MAR-1) by retroorbital injection for 3 days and underwent the PCA model. Some basophil-depleted mice underwent repletion with *St2*^*–/–*^BMBs or cells derived from heterozygous littermate controls 1 hour before challenge. Ear thickness was measured at 0, 1, and 24 hours after challenge. *P ≤ 0.05, **P ≤ 0.01, and ***P ≤ 0.001 (one-way ANOVA). Data are from at least 4 independent experiments, and the mean ± SEM of n = 15–20 mice per group (C) are displayed.
(TIFF)

S1 Dataset Spreadsheet containing all raw data presented in this manuscript. (XLSX)

S1 Raw Image Raw image file for S1 Fig, panel A. (JPG)
