# Supplementary figures and images for: Allergic inflammation is initiated by IL-33–dependent crosstalk between mast cells and basophils
Source: PLoS One. 2020 Jan 15;15(1):e0226701. doi: 10.1371/journal.pone.0226701 (PMC6961911; doi:10.1371/journal.pone.0226701)

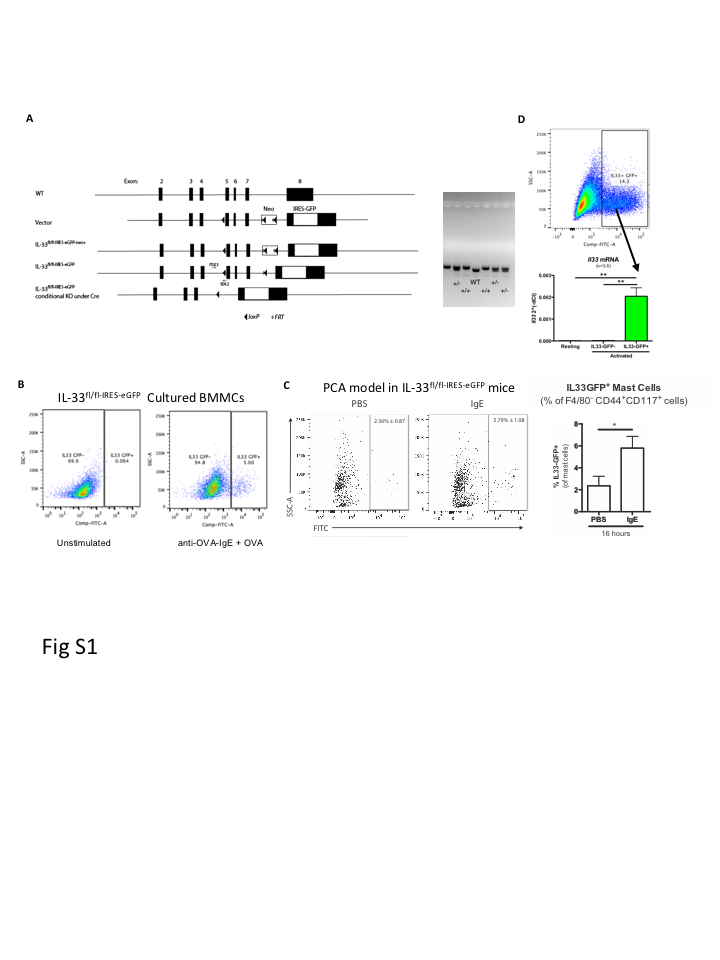

Supplement: S1 Fig — (A) Design and construction of the conditional Il33fl/fl-IRES-eGFP knock-in targeting vector. (B) BMMCs were generated from Il33fl/fl-IRES-eGFP and checked for purity as described in the methods. For IgE-mediated activation, BMMCs were coated in vitro with ovalbumin-specific IgE overnight and activated with ovalbumin (OVA) for 24 hours. Cells were harvested and analyzed by flow cytometry. IL33-GFP+ cells (% of live cells) is reported in the representative flow cytometry figures. (C) Il33fl/fl-IRES-eGFP mice underwent the PCA model with DNP-IgE/DNP-HSA as described in the methods. Ears were harvested 16 hours after challenge and digested for flow cytometry. IL33-GFP+ cells (of live F4/80–CD44+CD117+ mast cells) were identified by flow cytometry and quantified. (D) BMMCs from the Il33fl/fl-IRES-eGFP were activated as described in (B). GFP−and GFP+ cells were flow sorted and harvested for RNA. Il33 mRNA levels were quantified by RT-PCR. *P ≤ 0.05 and **P ≤ 0.01 (one-way ANOVA). Data are from at least 2 independent experiments, and the mean ± SEM of n = 7 (C) and n = 3–5 mice per group are displayed. (TIFF) [file pone.0226701.s001.tiff]

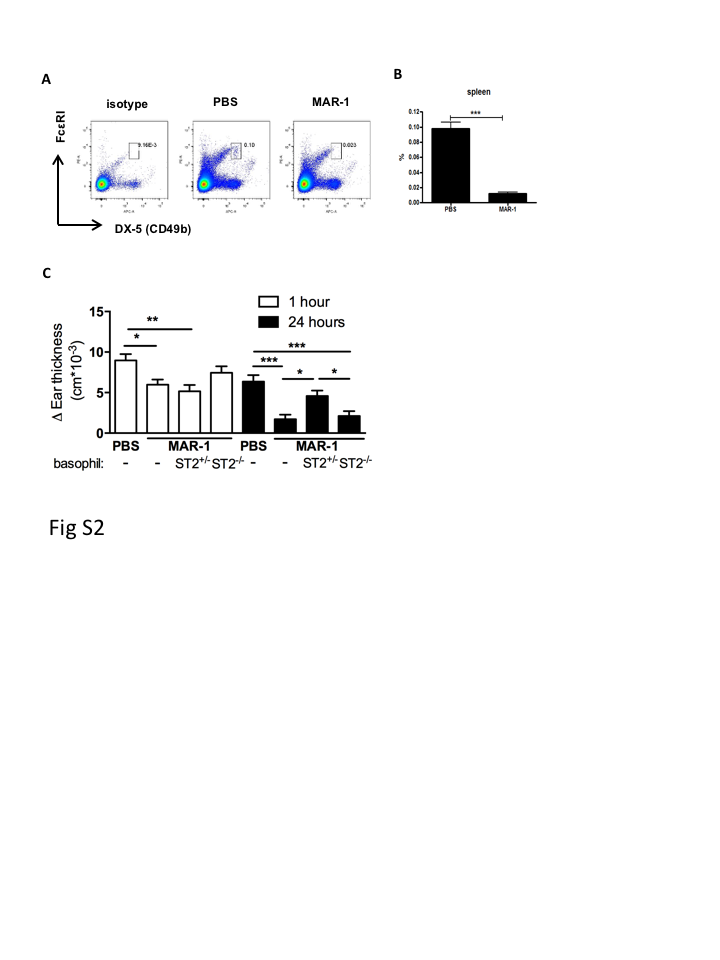

Supplement: S2 Fig — (A, B) C57BL/6J mice were retroorbitally injected with 10 μg anti-mFcεRI or PBS for 3 constitutive days. Representative flow data (A) and quantification (B) of spleen basophil population (CD49b+FcεRI+). Data is represented as mean ± SEM of n = 5 per group. ***P ≤ 0.001 (two-tailed Student’s t test). (C) C57BL/6J mice received PBS or 10 μg anti-mFcεRI (MAR-1) by retroorbital injection for 3 days and underwent the PCA model. Some basophil-depleted mice underwent repletion with St2–/–BMBs or cells derived from heterozygous littermate controls 1 hour before challenge. Ear thickness was measured at 0, 1, and 24 hours after challenge. *P ≤ 0.05, **P ≤ 0.01, and ***P ≤ 0.001 (one-way ANOVA). Data are from at least 4 independent experiments, and the mean ± SEM of n = 15–20 mice per group (C) are displayed. (TIFF) [file pone.0226701.s002.tiff]

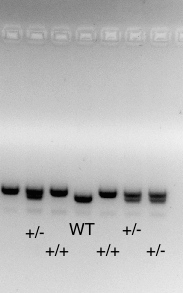

Supplement: S1 Raw Image — (JPG) [file pone.0226701.s004.jpg]
